# Mindfulness and its efficacy for psychological and biological responses in women with breast cancer

**DOI:** 10.1002/cam4.1052

**Published:** 2017-04-18

**Authors:** Elisabeth Kenne Sarenmalm, Lena B Mårtensson, Bengt A Andersson, Per Karlsson, Ingrid Bergh

**Affiliations:** ^1^ Research and Development CentreSkaraborg HospitalSkövdeSweden; ^2^ School of Health and EducationUniversity of Skövde; SkövdeSweden; ^3^ Department of Clinical immunology and transfusion medicineSahlgrenska University HospitalGothenburgSweden; ^4^ Department of OncologyInstitute of Clinical SciencesSahlgrenska AcademySahlgrenska University HospitalSweden University of GothenburgGothenburgSweden

**Keywords:** Breast cancer, immune response, mindfulness‐based stress reduction, randomized clinical trial

## Abstract

Many breast cancer survivors have to deal with a variety of psychological and physiological sequelae including impaired immune responses. The primary purpose of this randomized controlled trial was to determine the efficacy of a mindfulness‐based stress reduction (MBSR) intervention for mood disorders in women with breast cancer. Secondary outcomes were symptom experience, health status, coping capacity, mindfulness, posttraumatic growth, and immune status. This RTC assigned 166 women with breast cancer to one of three groups: MBSR (8 weekly group sessions of MBSR), active controls (self‐instructing MBSR) and non‐MBSR. The primary outcome measure was the Hospital Anxiety and Depression Scale. Secondary outcome measures were: Memorial Symptom Assessment Scale, SF‐36, Sense of Coherence, Five Facets of Mindfulness Questionnaire, and Posttraumatic Growth Index. Blood samples were analyzed using flow cytometry for NK‐cell activity (FANKIA) and lymphocyte phenotyping; concentrations of cytokines were determined in sera using commercial high sensitivity IL‐6 and IL‐8 ELISA (enzyme‐linked immunosorbent assay) kits. Results provide evidence for beneficial effects of MBSR on psychological and biological responses. Women in the MBSR group experienced significant improvements in depression scores, with a mean pre‐MBSR HAD‐score of 4.3 and post‐MBSR score of 3.3 (*P *=* *0.001), and compared to non‐MBSR (*P *=* *0.015). Significant improvements on scores for distress, symptom burden, and mental health were also observed. Furthermore, MBSR facilitated coping capacity as well as mindfulness and posttraumatic growth. Significant benefits in immune response within the MBSR group and between groups were observed. MBSR have potential for alleviating depression, symptom experience, and for enhancing coping capacity, mindfulness and posttraumatic growth, which may improve breast cancer survivorship. MBSR also led to beneficial effect on immune function; the clinical implications of this finding merit further research.

## Introduction

Although there have been enormous improvements in breast cancer diagnosis and advances in treatment, less attention has been paid to alleviating patients' breast cancer experience by preserving their physical, functional, and psychosocial well‐being 1. Women with breast cancer are challenged to cope over time with a high symptom burden and distress, which affects their well‐being and quality of life 2, 3, 4, 5, 6, 7. The prevalence of mood disorders is highest in the first year after breast cancer diagnosis and then decreases gradually over time 8.

Still, individuals report persistent coexistent physical and psychological symptoms that contribute to interference with daily life after breast cancer treatment 9.

An increasing body of research has established an association between distress and changes in immune function 10, 11. Distress seems to have a significant negative effect on immune function, such as lowered natural killer cells (NK cells) and T lymphocytes (T cells) 11, 12. T cells have been linked to breast cancer recurrence and survival 13, 14, 15. Other important parameters are cytokines, such as interleukin‐6 (IL‐6) and interleukin‐8 (IL‐8), which are independently correlated with breast cancer disease stage and progression 16, 17, 18, 19.

Thus, previous research indicates a significant need for interventions to improve well‐being, alleviate distress and symptom burden, and to reinforce immunity in women during breast cancer diagnosis, treatment, and recovery.

Originating from ancient Buddhist and yoga traditions, mindfulness‐based interventions have become increasingly popular in the Western world. Mindfulness is described as a “way of being” and defined as the capacity for awareness in each moment, by “paying attention in a particular way: on purpose, in the present moment, and nonjudgmentally”^20^. The use of mindfulness‐based interventions in oncology has proliferated over the past decade and research in the field has rapidly expanded 21, 22 While there appears to be evidence to support the use of mindfulness‐based interventions with cancer patients, the overall quality of existing trials varies considerably 23.

Mindfulness‐based stress reduction (MBSR) is an 8‐week, standardized program combining mindfulness meditation, yoga and other techniques designed to reduce stress and improve well‐being and quality of life in patients with a wide range of chronic pain and stress disorders 20, 24, 25. MBSR and has also been shown to improve mood disorders 21, 26and reduce stress in cancer patients 27, 28. Furthermore, MBSR reduces fear of recurrence and improves physical functioning which in turn leads to reduced stress and anxiety in women with breast cancer 29. Evidence from nonrandomized, uncontrolled studies suggests that MBSR improves quality of life and coping, decreases stress and alters cortisol and immune patterns 30, 31, 32, 33.

These results raise important questions as to whether MBSR is related to positive outcomes in mood disorders, symptom burden and health status, as well as strengthened immune system functioning in breast cancer survivors. Despite growing evidence that MBSR influences immune function, there is a need for studies to determine how biomarkers relate to changes in mindfulness and psychosocial outcomes, including symptom reduction and well‐being 34. While noting the effectiveness of MBSR, authors of several reviews have pointed out the inherent methodological problems in the published studies 35, 36. There is also a need for randomized controlled studies with long‐term follow‐up 36.

The primary purpose of our study was to determine the efficacy of MBSR intervention for mood disorder symptom improvements in women with breast cancer. Secondary goals were to evaluate their symptom experience, distress, health status, coping capacity, mindfulness, posttraumatic growth, and immune status.

## Patients and Methods

### Study design

In this 3‐month follow‐up study, we present the first results of a 5‐year longitudinal, randomized, controlled trial (RTC). Details of this trial have been described elsewhere 37.

The trial was designed in accord with Consort recommendation 38, 39, 40. In an unblinded RTC, participants' expectations about the intervention may lead to a placebo effect in the intervention group and/or a negative response among controls. In order to minimize a potential placebo effect in the active intervention group and a “frustrebo response”^41^ in controls, a three‐armed design was chosen.

Patients diagnosed with breast cancer were consecutively recruited to participate after completion of adjuvant chemotherapy and/or radiation therapy, with or without endocrine therapy. Patients were excluded on the basis of having another advanced illness at diagnosis that might interfere with the ability to participate, ongoing major depression, ongoing Herceptin therapy, or who had previously, as well as during the intervention, used MBSR and other mind‐body programs (including yoga).

This trial was approved by the Regional Ethical Review Board, University of Gothenburg, and informed consent was obtained before enrolment.

### Procedures

Eligible patients were contacted by research nurses at the first follow‐up appointment for patients receiving hormonal therapy or at the last treatment for patients undergoing chemotherapy. After oral and written information, interested patients provided written consent to participate in the study. Participants were first invited to a baseline health check‐up appointment, which included blood sample collection and questionnaire completion. Randomization was computerized and conducted in blocks of 9, 12, and 15, varied randomly. Assignment codes were kept in sequentially numbered, opaque, sealed envelopes, prepared by the research coordinator.

### Intervention

Participants were randomized into one of three groups:

MBSR (8 weeks self‐instructing MBSR program + instructor and weekly group sessions), active controls (8 weeks self‐instructing MBSR program) or non‐MBSR (no intervention).

Participants in the MBSR group attended a standardized, group‐based, 8‐week course once a week for an average of 2 h each week with homework assignments consisting of 20 min sessions, 6 days/week. Participants were provided with information material, including a 20‐page introduction to mindfulness training, a compacted disk (CD) with meditation exercises, the training program and a diary in order to report the time allotted to mindfulness training including patients′ reflections about the meditation exercises. Led by a certified MBSR instructor, these weekly group sessions focused on the participants' experiences of mindfulness, and including gentle meditation and yoga training. Active controls received information material, a CD, 8 weeks of self‐instructing training program and a diary. The only difference between MBSR group and active controls was the weekly group sessions. Participants in both MBSR group and active controls were provided with written and oral instructions how to use information material, CD and diary. All participants received standard care for follow‐up for breast cancer according to the national and local guidelines 42.

### Measures

Socio‐demographic data were collected through chart review and interviews. Clinical characteristics, patient self‐reported outcomes and biomarkers were collected at health checks both pre and postintervention. Follow‐ups for MBSR group and active controls were conducted 1 month after the intervention, and at similar time points. The same procedures, at similar time points of 3 months were conducted for those in the non‐MBSR group.

### Primary outcome measures

#### Mood disorder

Mood disorder was measured using the Hospital Anxiety and Depression scale (HAD), which is one of most widely used instruments to screen for anxiety and depression in cancer patients 43, 44, 45. The HAD is a 14‐item questionnaire consisting of two subscales: anxiety and depression. Subscale scores range from 0 to 21; scores for each subscale are defined as: 0–7 (normal), 8–10 (possible cases), and 11–21 (cases of psychological morbidity) 46. The internal consistency of reliability for both subscales are satisfactory, with Cronbach's alpha 0.72–0.89, respectively, 0.78–0.93 45

### Secondary outcomes measures

#### Symptom experience

Symptom experience was evaluated using the Memorial Symptom Assessment Scale (MSAS), a questionnaire consisting of 32 symptoms and symptom frequency, severity, and distress 47. The MSAS generates two subscales including physical and psychological symptoms, and two global indicators: Total Symptom Burden Scale (TMSAS) and the Global Symptom Distress Index (GDI). The MSAS is a reliable and valid multidimensional measure of symptom experience in cancer populations 47, 48 including the Swedish version of the MSAS 49.

#### Health status

Health status was measured using the 36‐item Short Form Health Survey (SF‐36), which consists of eight scaled scores: vitality, physical functioning, bodily pain, general health perceptions, physical, emotional and social role functioning, and mental health. The maximum score is 100 points. Reliability measurements of the SF‐36 are consistently good 50, 51, 52, 53.

#### Coping capacity

Coping capacity was evaluated using the Sense of Coherence scale (SOC) 54, 55, which consists of a 7‐point Likert scale evaluating perceived comprehensibility (5 items), manageability (4 items) and meaningfulness (4 items). Higher scores represent stronger sense of coherence. Reliability and validity of the SOC scale have been demonstrated, with Cronbach's *α* ranging from 0.74 to 0.93 56, 57, 58.

#### Mindfulness

Mindfulness was measured using the 29‐item short form Five Facets of Mindfulness Questionnaire (FFMQ–Swedish version), consisting of five key facets of mindfulness: observing, describing, acting with awareness, nonjudging, and nonreactivity to inner experience 59, 60.

#### Personal growth

Personal growth was evaluated using the Posttraumatic Growth Inventory (PTGI), which measures positive life changes after traumatic events. The PTGI yields a total score based on five dimensions: relating to others, new possibilities, personal strength, spiritual change, and appreciation of life 61. The PTGI has shown good reliability in previous research with a total score Cronbach's *α* of 0.96 62.

#### Lymphocyte distribution in peripheral blood

Lymphocyte distribution in peripheral blood was analyzed by flow cytometry using a FACSCanto II flow cytometer and the FACSDiva software. The absolute number of blood lymphocytes was determined with Trucount reference beads using the method recommended by the manufacturer. The following subpopulations were reported CD3^+^, CD3^+^4^+^ and CD3^+^8^+^T cells, CD19^+^B cells, and CD3‐16^+^56^+^NK cells. The results for each subpopulation were expressed as the percentage of lymphocytes and as the number of cells × 10^9^/L. Antibodies to the antigens above. Trucount beads, the FACSCanto II flow cytometer and the FACSDiva software were all from BD Biosciences, Mountain View, CA.

#### NK‐cell activity

NK‐cell activity was measured using a Flow‐cytometric Assay of Natural Killer cell Immune response in Activated whole blood (FANKIA) a modified version of a previously published method using flow cytometry and stained K562 cells as target cells 63. Whole blood was mixed with a defined number of target cells transfected with the gene for green fluorescent protein (GFP) 64. After incubation the same volume was collected from tubes with: blood and target cells, target cells and medium; and blood and medium and analyses were performed as described above. The lytic activity was defined as the reduction in the number of target cells after mixing with the blood, expressed in percentage of target cells.

#### Determination of cytokine concentrations

Determination of cytokine concentrations were determined in sera using commercial high sensitivity IL‐6 and IL‐8 ELISA kits (R&D Systems, Inc., Abingdon, UK) according to the instructions from the manufacturer.

### Data analysis

Sample size calculation was based on the primary outcome: breast cancer patient's mood disorder symptoms. A one‐unit change on the HAD‐subscales from baseline to 3‐month follow‐up was regarded as clinically relevant. The detection of such a difference would require 50 participants per group (a total of 150 participants) to achieve a statistical power of 80%. Descriptive statistics were used to summarize socio‐demographic and clinical characteristics. Spearman's correlation coefficients were calculated to determine the strength of relationships between selected variables. As most of the variables we explored were of ordinal data type and most of the continuous variables deal with skewed distributions deviating from normal‐distribution, we used nonparametric tests (Wilcoxon's test for comparison within groups and Mann–Whitney's test for comparison between groups).

*P* < 0.05 was considered as statistically significant result.

## Results

A total of 177 women consented participation and were randomly assigned to one of three groups. There were 11 drop‐outs after randomization, that is, two patients were excluded as they did not complete the intervention, two patients withdraw their participation due to rapid breast cancer disease progression, and seven patients did not visited first follow‐up (MBSR* *=* *4; active controls* *=* *5; non‐MBSR* *=* *2).

**Figure 1:**
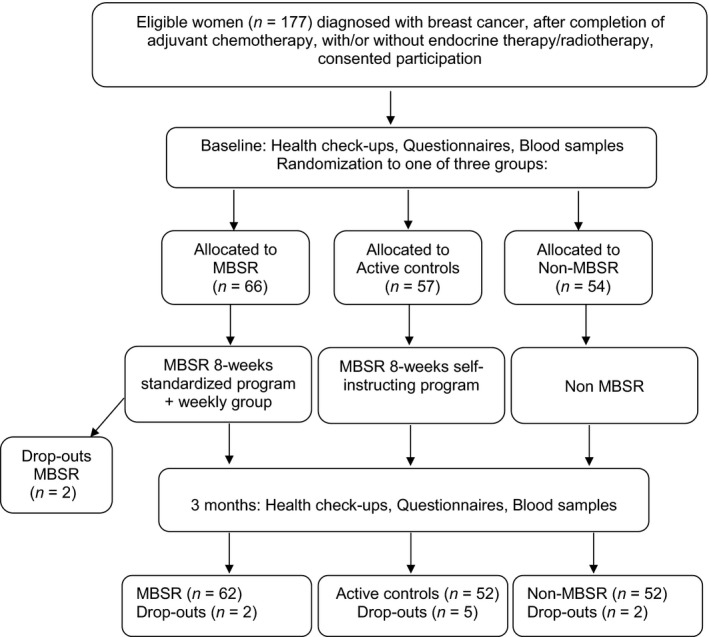
Flowchart of study design and randomization.

The final groups were MBSR (*n *=* *62), active controls (*n *=* *52) and non‐MBSR (*n *=* *52). Postintervention data were missing for one active control participant. A participation flowchart is depicted in Figure 1.

Participants' ranged from 34 to 80 years (mean* *=* *57.2, SD* *=* *10.2). No statistical differences were found between groups on demographic or clinical characteristics. Participant descriptions are listed in Table 1.

**Table 1:** Baseline demographic data and clinical characteristics

Characteristic	MBSR group (*n* = 62)		Active controls (*n* = 52)		Non‐MBSR (*n* = 52)		Total (*n* = 166)	
	*N*	%	*N*	%	*N*	%	*N*	%
Marital status								
Married/cohabitation	46	74.2	35	67.3	42	80.8	123	74.1
Widowed	3	4.8	3	5.8	4	7.7	10	6.0
Divorced	6	9.7	8	15.4	1	1.9	15	9.0
Single	5	8.1	4	7.7	4	7.7	13	7.8
Partner without l.t.^a^	2	3.2	2	3.8	1	1.9	5	3.0
Living with								
Partner	44	71.0	35	67.3	41	78.8	120	72.3
Other	1	1.6	0	0	1	1.9	2	1.2
Children	7	11.3	2	3.8	2	3.8	11	6.6
Living alone	10	16.1	15	28.8	8	15.4	33	19.9
Education								
Primary school	5	8.1	1	1.9	1	1.9	7	4.2
Secondary school	14	22.6	10	19.2	11	21.2	35	21.1
Add education–lower^b^	6	9.7	6	11.5	10	19.2	22	13.3
Add education– higher^c^	7	11.3	11	21.2	5	9.6	23	13.9
University	30	48.4	24	46.2	25	48.1	79	47.6
Children								
Yes	57	91.9	49	94.2	45	86.5	151	91.0
No	5	8.1	3	5.8	7	13.5	15	9.0
Employment status								
Working	38	63.3	38	74.5	35	68.6	111	68.5
Unemployed	1	1.7	0	0	0	0	1	0.6
Disability pensioner	2	3.3	3	5.9	1	2.0	6	3.7
Retired	18	30.0	10	19.6	15	29.4	43	26.5
Other	1	1.7	0	0	0	0	1	0.6
Surgery								
Mastectomy	30	48.4	26	50.0	21	40.4	77	46.4
Lumpectomy	33*	53.2	26	50.0	30	57.7	89	53.6
Other	0	0	0	0	1	1.9	1	0.6
Tumor size								
<2 cm	28	45.9	25	49.0	32	64.0	85	52.5
2–5 cm	25	41.0	22	43.1	8	16.0	55	34.0
>5 cm	8	13.1	4	7.8	10	20.0	22	13.6
Type of cancer								
Ductal	43	69.4	37	71.2	40	76.9	120	72.3
Lobular	13	21.0	9	17.3	6	11.5	28	16.9
Other	6	9.7	6	11.5	6	11.5	18	10.8
Receptor								
ER+/PgR+	40	69.0	34	72.3	39	78.0	113	72.9
ER+/PgR‐	8	13.8	6	12.8	4	8.0	17	11.6
ER‐/PgR+	0	0.0	0	0.0	1	2.0	1	0.6
ER‐/PgR‐	10	17.2	7	14.9	6	12.0	24	14.8
BRE (mean)	6.6		6.5		6.6			
Treatment postop								
Chemotherapy (CT)	2	3.2	3	5.8	3	5.8		
Radiotherapy (RT)	3	4.8	5	9.6	4	7.7		
Hormonal therapy (HT)	12	19.4	14	26.9	6	11.5		
CT+RT	11	17.8	6	11.5	7	13.5		
CT+ HT	4	6.5	0	0	1	1.9		
RT+ HT	16	25.8	13	25.0	16	30.8		
CT+RT+HT	13	20.9	10	19.2	14	26.9		
No treatment	1	1.6	1	1.9	1	1.9		

ER, estrogen receptor; PgR, progesterone receptor.

a Partner, not living together with.

b Lower additional education.

c Higher additional education.

### Psychological response

Study results revealed significant changes in psychological and biological responses to the MBSR intervention, summarized in Tables 2, 3, 4, 5, 6, 7, 8.

**Table 2:** Primary outcome: pre and postintervention mood disorder symptoms

Measures HAD^a^	Group	Preintervention		Postintervention		*P*‐value^b^	*P*‐value^c^
		Mean (SD)	Median (min‐max)	Mean (SD)	Median (min‐max)		
Anxiety	MBSR	6.5 (4.3)	6 (0–19)	6.0 (3.9)	6 (0–16)	0.069	0.080
	Active Controls	5.6 (3.9)	4.5 (0–14)	5.1 (3.9)	5 (0–13)	0.236	0.109
	Non‐MBSR	4.8 (3.6)	4.5 (0–12)	5.5 (4.1)	5 (0–15)	0.355	Ref.
Depression	MBSR	4.3 (3.7)	4 (0–14)	3.3 (3.3)	2 (0–12)	**0.001** a	**0.015** a
	Active Controls	3.4 (3.4)	2 (0–14)	3.0 (2.9)	2 (0–12)	0.292	0.472
	Non‐MBSR	3.5 (3.5)	2 (0–14)	3.8 (3.8)	2 (0–15)	0.892	Ref.

Significant changes are marked in bold.

a The Hospital Anxiety and Depression Scale (HAD).

b Change over time (*P*‐value) within groups (Wilcoxon signed test).

c Change over time (*P*‐value) between groups (comparison of MBSR‐Active controls vs. Non‐MBSR (ref.) (Mann–Whitney test).

**Table 3:** Secondary outcome: symptom experience

Measure MSAS^a^	Group	Preintervention		Postintervention		*P‐*value^b^	*P*‐value^c^
		Mean (SD)	Median (min‐max)	Mean (SD)	Median (min‐max)		
Physical symptoms	MBSR	0.7 (0.5)	0.61 (0–2.30)	0.6 (0.4)	0.52 (0–2.15)	**0.007** a	0.245
	Active controls	0.5 (0.4)	0.49 (0–1.45)	0.5 (0.4)	0.41 (0–2.13)	0.372	0.966
	Non‐MBSR	0.6 (0.5)	0.46 (0–2.11)	0.5 (0.5)	0.45 (0–2.34)	0.475	Ref.
Psychological symptoms	MBSR	1.4 (0.8)	1.34 (0–3.22)	1.2 (0.9)	0.98 (0–3.32)	**0.008** a	**0.019** a
	Active controls	1.0 (0.9)	0.77 (0–2.77)	0.9 (0.8)	0.69 (0–2.67)	0.335	0.337
	Non‐MBSR	0.9 (0.8)	0.76 (0–2.92)	0.9 (0.8)	0.83 (0–2.82)	0.800	Ref.
Global distress	MBSR	1.9 (0.6)	1.93 (0–2.97)	1.8 (0.6)	1.80 (0–3.05)	0.054	**0.013** a
	Active controls	1.7 (0.8)	1.80 (0–3.24)	1.6 (0.8)	1.60 (0–3.20)	0.121	**0.015** a
	Non‐MBSR	1.6 (0.8)	1.79 (0–4.0)	1.7 (0.9)	2.0 (0–3.2)	0.103	Ref.
Total symptom burden	MBSR	0.8 (0.5)	0.71 (0–2.13)	0.7 (0.5)	0.55 (0–2.22)	**0.004** a	0.097
	Active controls	0.6 (0.4)	0.56 (0–1.52)	0.5 (0.3)	0.49 (0–1.5)	0.113	0.671
	Non‐MBSR	0.6 (0.4)	0.54 (0–1.59)	0.6 (0.4)	0.56 (0.05–2.0)	0.379	Ref.


Significant changes are marked in bold.

a MSAS, Memorial Symptom Assessment Scale.

b Change over time (*P*‐value) within groups (Wilcoxon signed test).

c Change over time (*P*‐value) between groups (comparison of MBSR‐Active controls vs. Non‐MBSR (ref.) (Mann–Whitney test).

**Table 4:** Secondary outcome: health status

Measure SF‐36^a^	Group	Preintervention		Postintervention		*P*‐value^b^	*P*‐value^c^
		Mean (SD)	Median (min‐max)	Mean (SD)	Median (min‐max)		
Vitality	MBSR	49.5 (27.5)	52.5 (29–75)	60.9 (20.1)	65 (45–76)	**0.000** a	0.159
	Active Controls	56.9 (24.4)	60 (40–75)	62.5 (23.7)	65 (45–80)	0.129	0.838
	Non‐MBSR	55.0 (25.8)	55 (40–80)	58.3 (24.4)	60 (35–80)	0.397	Ref.
Physical functioning	MBSR	77.4 (16.7)	80 (67.5–90)	83.1 (15.4)	90 (75–95)	**0.000** a	0.822
	Active Controls	83.7 (17.3)	90 (75–95)	84.8 (18.5)	95 (80–95)	0.412	0.129
	Non‐MBSR	78.5 (19.6)	80 (70–95)	82.8 (19.0)	90 (71.0–95)	**0.007** a	Ref.
Bodily pain	MBSR	65.2 (26.4)	67 (45–90)	71.4 (23.5)	77.5 (45–92)	0.099	0.799
	Active Controls	70.9 (20.7)	77.5 (55–90)	74.4 (25.2)	79.5 (57–100)	0.466	0.526
	Non‐MBSR	70 (23.1)	67 (56–90)	73.5 (27.1)	85 (56–100)	0.253	Ref.
General health perceptions	MBSR	61.2 (21.6)	60 (45–75)	67.6 (19.1)	70 (52–85)	**0.003** a	0.087
	Active Controls	67.2 (19.3)	75 (55–80)	70.6 (19.1)	70 (60–85)	0.111	0.465
	Non‐MBSR	69.3 (19.4)	70 (60–85)	69.6 (19.6)	75 (55–80)	0.832	Ref.
Physical role functioning	MBSR	45.9 (42.9)	50 (0–100)	63.3 (39.7)	75 (25–100)	**0.003** a	0.822
	Active Controls	42.2 (42.6)	25 (0–100)	67.6 (35.8)	75 (50–100)	**0.001** a	0.463
	Non‐MBSR	40.4 (41.5)	25 (0–75)	59.6 (42.3)	75 (6–100)	**0.006** a	Ref.
Emotional role functioning	MBSR	61.7 (41.2)	67 (33–100)	72.1 (38.6)	100 (33–100)	0.053	0.900
	Active Controls	69.9 (38.5)	100 (33–100)	77.1 (35.6)	100 (67–100)	0.168	0.652
	Non‐MBSR	60.9 (47.0)	100 (0–100)	72.4 (37.2)	100 (33–100)	0.093	Ref.
Social functioning	MBSR	74.0 (23.0)	75 (62–100)	79.0 (20.7)	87.5 (62–100)	0.103	0.117
	Active Controls	78.4 (25.9)	87.5 (62–100)	84.8 (22.8)	100 (62–100)	**0.017** a	0.105
	Non‐MBSR	72.1 (26.0)	75 (50–100)	84.1 (25.3)	100 (75–100)	**0.001** a	Ref.
Mental health	MBSR	67.9 (19.0)	72 (56–80)	74.1 (17.1)	76 (64–85)	**0.000** a	**0.001** a
	Active Controls	73.5 (22.7)	80 (60–92)	77.8 (17.4)	80 (64–92)	0.073	**0.038** a
	Non‐MBSR	76.2 (20.0)	84 (64–88)	74.4 (20.7)	84 (60–92)	0.299	Ref.

Significant changes are marked in bold.

a 36‐items short form health survey (SF‐36).

b Change over time (*P*‐value) within groups (Wilcoxon signed test).

c Change over time (*P*‐value) between groups (comparison of MBSR‐Active controls vs. Non‐MBSR (ref.) (Mann–Whitney test).

**Table 5:** Secondary outcome: coping capacity

Measure SoC^a^	Group	Preintervention		Postintervention		*P*‐value^b^	*P*‐value^c^
		Mean (SD)	Median (min‐max)	Mean (SD)	Median (min‐max)		
Coping capacity	MBSR	65.7 (13.7)	67 (32–91)	67.7 (12.0)	66.5 (39–91)	0.055	**0.028** a
	Active controls	69.8 (13.7)	71 (28–90)	70.8 (12.5)	75 (41–90)	0.458	0.098
	Non‐MBSR	71.4 (11.1)	72 (39–88)	69.3 (11.5)	69 (45–87)	0.113	Ref.

Significant changes are marked in bold.

a SoC, Sense of Coherence.

b Change over time (*P*‐value) within groups (Wilcoxon signed test).

c Change over time (*P*‐value) between groups (comparison of MBSR‐Active controls vs. Non‐MBSR (ref.) (Mann–Whitney test).

**Table 6:** Secondary outcome: facets of mindfulness

Measure FFMQ^a^	Group	Preintervention		Postintervention		*P*‐value^b^	*P*‐value^c^
		Mean (SD)	Median (min‐max)	Mean (SD)	Median (min‐max)		
Nonreactivity	MBSR	2.9 (0.7)	3.0 (1.3–4.5)	3.3 (0.5)	3.2 (2.0–4.7)	**0.000** a	**0.010** a
	Active Controls	3.2 (0.6)	3.2 (1.8–4.3)	3.3 (0.5)	3.3 (1.8–4.3)	0.318	0.759
	Non‐MBSR	3.1 (0.6)	3.2 (1.0–4.3)	3.2 (0.7)	3.2 (1.0–4.3)	0.552	Ref.
Observe	MBSR	3.3 (0.7)	3.3 (1.0–4.7)	3.6 (0.5)	3.7 (1.7–4.9)	**0.000** a	**0.006** a
	Active Controls	3.3 (0.7)	3.3 (1.4–4.9)	3.4 (0.7)	3.4 (1.4–5.0)	**0.015** a	0.376
	Non‐MBSR	3.1 (0.7)	3.1 (1.6–4.1)	3.2 (0.7)	3.3 (1.0–4.6)	0.190	Ref.
Awareness	MBSR	3.3 (0.8)	3.4 (1.8–5.0)	3.3 (0.6)	3.2 (2.0–5.0)	0.751	0.783
	Active Controls	3.5 (0.8)	3.6 (1.8–5.0)	3.4 (0.7)	3.4 (2.0–5.0)	0.280	0.224
	Non‐MBSR	3.4 (0.7)	3.3 (1.8–4.8)	3.4 (0.8)	3.3 (2.0–5.0)	0.984	Ref.
Describe	MBSR	3.6 (0.7)	3.7 (2.0–5.0)	3.6 (0.6)	3.8 (2.0–4.7)	0.599	0.478
	Active Controls	3.6 (0.6)	3.7 (2.0–4.8)	3.6 (0.7)	3.7 (2.0–5.0)	0.938	0.700
	Non‐MBSR	3.5 (0.7)	3.7 (1.8–5.0)	3.5 (0.8)	3.6 (1.0–5.0)	0.659	Ref.
Nonjudge	MBSR	3.3 (0.8)	3.4 (1.2–5.0)	3.3 (0.8)	3.2 (1.6–5.0)	0.992	0.361
	Active Controls	3.5 (0.8)	3.4 (2.0–5.0)	3.4 (0.7)	3.4 (2.4–5.0)	0.339	0.775
	Non‐MBSR	3.7 (0.8)	3.6 (2.0–5.0)	3.5 (0.9)	3.6 (1.8–5.0)	0.164	Ref.

Significant changes are marked in bold.

a FFMQ, Five Facets of Mindfulness Questionnaire.

b Change over time (*P*‐value) within groups (Wilcoxon signed test).

c Change over time (*P*‐value) between groups (comparison of MBSR‐Active controls vs. Non‐MBSR (ref.) (Mann–Whitney test).

**Table 7:** Secondary outcome: Posttraumatic Growth

Measure PTGI^a^	Group	Preintervention		Postintervention		*P*‐value^b^	*P*‐value^c^
		Mean (SD)	Median (min‐max)	Mean (SD)	Median (min‐max)		
Posttraumatic growth	MBSR	59.78 (19.5)	62.00 (17–103)	64.65 (17.7)	67.00 (6–100)	**0.005**	0.111
	Active Controls	55.92 (20.2)	58.50 (0–92)	57.13 (17.6)	61.00 (17–89)	0.498	**0.049**
	Non‐MBSR	52.58 (19.2)	55.50 (13–85)	51.57 (20.8)	52.00 (0–94)	0.933	Ref.

Significant changes are marked in bold.

a PTGI, Posttraumatic Growth Inventory.

b Change over time (*P*‐value) within groups (Wilcoxon signed test).

c Change over time (*P*‐value) between groups (comparison of MBSR‐Active controls vs. Non‐MBSR (ref.) (Mann–Whitney test).

**Table 8:** Secondary outcome: immune response

Measures	Group	Preintervention		Postintervention		*P*‐value^a^	*P*‐value^b^
		Mean (SD)	Median (quartiles)	Mean (SD)	Median (quartiles)		
Fankia%	MBSR	19.1 (8.2)	18 (14–25)	22.0 (7.7)	22 (17–27)	**0.015**	0.142
	Active Controls	22.0 (7.6)	20 (14.5–26.5)	18.8 (7.3)	19 (16–23)	0.668	0.602
	Non‐MBSR	19.2 (8.4)	19 (13–24)	19.6 (6.8)	20 (15–25)	0.731	Ref
Lymphocytesx10e9/I	MBSR	1.5 (0.76)	1.3 (0.98–1.7)	1.4 (0.58)	1.4 (0.98–1.7)	0.997	0.559
	Active Controls	1.6 (0.7)	1.4 (1.2–1.9)	1.5 (0.5)	1.4 (1.1–1.7)	0.415	0.280
	Non‐MBSR	1.4 (0.6)	1.3 (0.96–1.7)	1.4 (0.6)	1.2 (0.97–1.8)	0.518	Ref
CD3T%	MBSR	72.8 (11.1)	74 (66.7–82.0)	71.4 (10.5)	72 (67–80)	**0.027**	0.222
	Active Controls	72.9 (7.5)	71 (68–78)	71.5 (7.7)	71 (68–77)	0.289	0.061
	Non‐MBSR	76.3 (8.1)	78 (71–82)	73.4 (8.8)	75 (67–79)	**0.001**	Ref
CD3Tx10e9/I	MBSR	1.1 (0.6)	0.97 (0.67–1.3)	1.0 (0.5)	1.0 (0.63–1.3)	0.671	0.800
	Active Controls	1.2 (0.6)	1.1 (0.8–1.4)	1.1 (0.4)	1.0 (0.7–1.2)	0.263	0.424
	Non‐MBSR	1.0 (0.4)	1 (0.8–1.3)	1.0 (0.5)	0.8 (0.7–1.3)	0.937	Ref
CD3^+^4^+^Th%	MBSR	41.7 (8.7)	42 (37–48)	41.1 (8.7)	40.5 (35–47)	0.274	0.253
	Active Controls	44.9 (8.1)	46 (39–50)	44.5 (8.4)	45 (41–50)	0.987	0.080
	Non‐MBSR	46.2 (9.8)	46 (38–55)	44.3 (9.1)	43.5 (39–50)	**0.021**	Ref
CD3^+^4^+^x10e9/I	MBSR	0.62 (0.31)	0.54 (0.4–0.7)	0.60 (0.29)	0.53 (0.4–0.7)	0.581	0.860
	Active Controls	0.73 (0.4)	0.69 (0.5–0.8)	0.66 (0.3)	0.65 (0.4–0.8)	0.543	0.864
	Non‐MBSR	0.63 (0.28)	0.62 (0.4–0.8)	0.62 (0.31)	0.56 (0.4–0.7)	0.834	Ref
CD3^+^8^+^T cy/s%	MBSR	29.6 (11.3)	28 (20–37)	28.4 (10.5)	25.5 (20–38)	**0.035**	1.00
	Active Controls	27.9 (10.4)	26 (21–35)	26.7 (9.5)	25 (20–34)	**0.041**	0.819
	Non‐MBSR	28.6 (11.2)	25 (21–38)	27.8 (11.7)	24.5 (19–36)	0.132	Ref
CD3^+^8^+^x10e9/I	MBSR	0.46 (0.33)	0.37 (0.2–0.6)	0.42 (0.26)	0.33 (0.2–0.6)	0.658	0.750
	Active Controls	0.44 (0.26)	0.38 (0.3–0.6)	0.38 (0.18)	0.34 (0.23–0.5)	0.144	0.384
	Non‐MBSR	0.40 (0.27)	0.32 (0.2–0.5)	0.41 (0.30)	0.28 (0.2–0.5)	0.655	Ref
CD3‐16^+^56^+^NK%	MBSR	16.7 (8.1)	14.5 (11–21)	16.1 (7.5)	15 (10–21)	0.329	0.077
	Active Controls	15.9 (6.1)	15 (12–20)	15.2 (6.2)	15 (11–19)	0.057	**0.025**
	Non‐MBSR	14.8 (6.2)	13.5 (10–19)	15.7 (7.1)	15.5 (10–18)	0.127	Ref
CD3‐16^+^56^+^NKx10e9/I	MBSR	0.24 (0.16)	0.2 0.1–0.3)	0.22 (0.10)	0.2 (0.1–0.3)	0.239	**0.041**
	Active Controls	0.25 (0.14)	0.24 (0.1–0.3)	0.22 (0.13)	0.19 (0.1–0.3)	0.121	**0.011**
	Non‐MBSR	0.20 (0.11)	0.17 (0.1–0.3)	0.22 (0.13)	0.18 (0.1–0.3)	0.051	Ref
CD19B%	MBSR	9.5 (8.4)	7.5 (3.7–13)	11.6 (7.6)	10 (7–14)	**0.001**	0.957
	Active Controls	10.2 (4.8)	10 (7–13)	12.2 (5.5)	12 (9–15)	**0.007**	0.417
	Non‐MBSR	7.7 (5.4)	8 (3–11)	9.8 (4.7)	9 (6–12)	**0.003**	Ref
CD19Bx10e9/I	MBSR	0.15 (0.16)	0.1 (0.04–0.21)	0.17 (0.15)	0.1 (0.08–0.21)	**0.004**	0.862
	Active Controls	0.17 (0.12)	0.14 (0.08–0.23)	0.17 (0.09)	0.17 (0.11–0.22)	0.385	0.362
	Non‐MBSR	0.11 (0.08)	0.1 (0.03–0.16)	0.13 (0.07)	0.13 (0.09–0.17)	**0.013**	Ref
CD3+4+/CD3+8+quotient%	MBSR	1.65 (0.73)	1.6 1–2.2)	1.72 (0.82)	1.6 (1–2.2)	0.220	0.313
	Active Controls	1.92 (0.99)	1.7 (1.2–2.3)	1.99 (1.1)	1.8 (1.2–2.3)	0.272	0.336
	Non‐MBSR	1.98 (1.2)	1.7 (1.1–2.6)	2.06 (1.7)	1.8 (1.1–2.5)	0.812	Ref
IL6‐HS (ELISA)	MBSR		1.0 (0.7–2.3)		1.0 (0.6–1.7)	0.750	0.847
	Active Controls		1.2 (0.7–1.7)		1.0 (0.7–2.1)	0.983	0.528
	Non‐MBSR		1.1 (0.5–2.5)		1.2 (0.5–1.8)	0.291	Ref.
IL8‐HS (ELISA)	MBSR		11.5 (9.3–5)		12.0 (9.7–15.7)	0.241	0.395
	Active Controls		12.0 (9.0–4)		12.0 (9.4–15.0)	1.000	0.595
	Non‐MBSR		12.0 (9.0–5)		12.0 (9.3–16.0)	0.894	Ref.

Significant changes are marked in bold.

a Change over time (*P*‐value) within groups (Wilcoxon signed test).

b Change over time (*P*‐value) between groups (comparison of MBSR‐Active controls vs. Non‐MBSR (ref.) (Mann–Whitney test).

On the primary outcome measures, MBSR participants reported significant improvements on depression symptoms both within group (mean* *=* *4.3; SD* *=* *3.7 to mean* *=* *3.3; SD* *=* *3.3; *P *=* *0.001) as well as compared to non‐MBSR (*P *=* *0.015**)**, but not on anxiety symptoms (Table 2).

The number of reduced cases of depression were 7 (11%) in MBSR participants compared to 4 (8%) reduced cases of depression in active controls and non‐MBSR, respectively.

**Figure 2:**
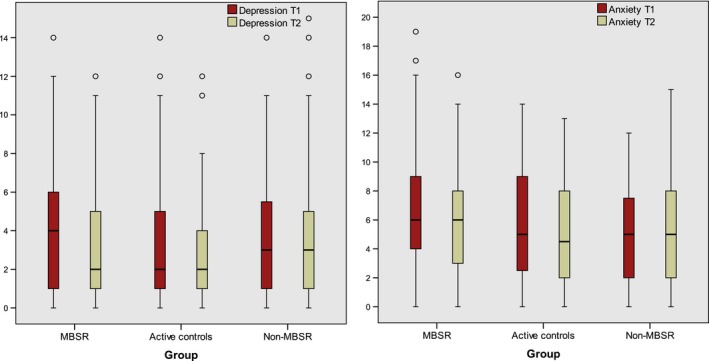
Box‐plot. Primary outcome: Pre and Postintervention Mood Disorder Symptoms, HAD scores for depression, respectively, anxiety subscales.

Pre and postintervention HAD scores for depression and anxiety are presented in Figure 2.

There were significant improvements in physical symptoms (mean* *=* *0.7; SD* *=* *0.5 to mean* *=* *0.6; SD* *=* *0.4; *P *=* *0.007), psychological symptoms (mean* *=* *1.4; SD* *=* *0.8; to mean* *=* *1.2; SD* *=* *0.9; *P *=* *0.008), and total symptom burden (mean* *=* *0.8; SD* *=* *0.5 to mean* *=* *0.7; SD* *=* *0.5; *P *=* *0.004) within the MBSR. There were also significant improvements between MBSR versus non‐MBSR regarding psychological symptoms (*P *=* *0.019), as well as global distress (*P *=* *0.013) (Table 3).

Within the MBSR group, changes in health status were seen in improved vitality (mean* *=* *49.5; SD* *=* *27.5 to mean* *=* *60.9; SD* *=* *20.1; *P *<* *0.001), physical functioning (mean* *=* *77.4; SD* *=* *16.7 to mean* *=* *83.1; SD* *=* *15.4; *P *<* *0.001), general health perceptions (mean* *=* *61.2; SD* *=* *21.6 to mean* *=* *67.6; SD* *=* *19.1; *P *=* *0.003), physical role functioning (mean* *=* *45.9; SD* *=* *42.9 to mean* *=* *63.3; SD* *=* *39.7; *P *=* *0.003), and mental health (mean* *=* *67.9; SD* *=* *19.0 to mean* *=* *74.1; SD* *=* *17.1; *P *<* *0.001). Changes were reported in physical functioning within the non‐MBSR group (mean* *=* *78.5; SD* *=* *19.6 to mean* *=* *82.8; SD* *=* *19.0; *P *=* *0.007), physical role functioning with active controls (mean* *=* *42.2; SD* *=* *42.6 to mean* *=* *67.6; SD* *=* *35.8; *P *=* *0.001), non‐MBSR (mean* *=* *40.4; SD* *=* *41.5 to mean* *=* *59.6; SD* *=* *42.3; *P *=* *0.006), social functioning for active controls (mean* *=* *78.4; SD* *=* *25.9 to mean* *=* *84.8; SD* *=* *22.8; *P *=* *0.017), non‐MBSR (mean* *=* *72.1; SD* *=* *26.0 to mean* *=* *84.1; SD* *=* *25.3; *P *=* *0.001). A significant improvement between groups was reported in mental health for the MBSR group (*P *=* *0.001) and active controls (*P *=* *0.038) compared to non‐MBSR (Table 4).

Additional secondary outcome measures showed that MBSR participants reported improved coping capacity (*P *=* *0.028) versus non‐MBSR (Table 5).

Enhanced elements of mindfulness were shown regarding Nonreactivity within MBSR (mean* *=* *2.9; SD* *=* *0.7 to mean* *=* *3.3; SD* *=* *0.5; *P *<* *0.001), and between groups compared to non‐MBSR (*P *=* *0.010), as well as on Observe both within MBSR group (mean* *=* *3.3; SD* *=* *0.7 to mean* *=* *3.6; SD* *=* *0.5; *P *<* *0.001) and compared to non‐MBSR (*P *=* *0.006) (Table 6). Enriched posttraumatic growth was reported within MBSR (mean* *=* *59.78; SD* *=* *19.5 to mean* *=* *64.65; SD* *=* *17.7; *P *=* *0.005), and between groups for active controls versus non‐MBSR (*P *=* *0.049) (Table 7).

### Biological response

Mean baseline NK‐cell activity increased significantly (mean* *=* *19.1; SD* *=* *8.2 to mean* *=* *22.0 SD* *=* *7.7; *P *=* *0.015) within the MBSR group. The absolute number of CD19^+^B‐lymphocytes increased (mean* *=* *0.15; SD* *=* *0.16 to mean* *=* *0.17; SD* *=* *0.15; *P *=* *0.004). The proportion of CD3^+^T‐lymphocytes decreased (mean = 72.8; SD = 11.1 to mean = 71.4; SD = 10.5; *P* = 0.027) as did that of CD3^+^8^+^T‐lymphocytes (mean* *=* *29.6; SD = 11.3 to mean = 28.4; SD = 10.5; *P* = 0.035), whereas the proportion of CD19^+^B‐lymphocytes increased (mean = 9.5; SD = 8.4 to mean = 11.6; SD = 7.6; *P* = 0.001). Analyses also demonstrated significant changes (*P *=* *0.041) between MBSR participants and non‐MBSR regarding decrease in the absolute number of NK cells (CD3‐16^+^56^+^NKx10e9/l).

There were also some significant changes pre and postintervention in active controls and in the non‐MBSR group suggesting a decrease in the absolute number of NK cells (CD3‐16‐56^+^NKx10e9/I) for active controls (*P *=* *0.011) compared to non‐MBSR. There were also significant changes for active controls regarding CD3‐16^+^56^+^NK% versus non‐MBSR (*P *=* *0.025) (Table 8). There were no significant differences in serum concentrations of IL‐6 or IL‐8 between any of the study groups.

## Discussion

Our trial provides evidence in support of the efficacy of MBSR for psychological and biological response among women with breast cancer. The primary purpose of this study was to determine the efficacy of MBSR intervention on mood disorder, that is, depression and anxiety. Our finding of improvements in depression is consistent with other RTCs that have evaluated MBSR and mood disorders in breast cancer patients 21, 26. Unlike those studies, our findings revealed no significant changes in anxiety. However, consistent with our trial, a meta‐analysis of mindfulness‐based interventions that included participants who met the diagnostic criteria for a current episode of anxiety or depressive disorder show that MBSR is effective for reducing symptoms of depression, but not anxiety 65.

MBSR participants reported significantly greater improvements in symptoms, especially psychological symptoms. In addition, their symptom burden and distress significantly decreased. MBSR participants also improved in functional status; in line with previous research showing significant intergroup improvements in mental health 66, our findings indicate significant improved mental health between groups.

A common assumption is that mindfulness increases the individual's ability to cope, but few RCTs have examined the effect of MBSR on coping capacity. The MBSR intervention appears to improve coping effectiveness in breast cancer patients 32, and behavioral and cognitive coping 67. Results from our trial show that women who participated in the MBSR experienced improved coping capacity, here measured as sense of coherence (SOC). Previous research has identified SOC as a significant predictor of distress, number and type of coping strategies in women with breast cancer 68, suggesting the lower the SOC, the higher the levels of symptom burden 3. While Antonovsky 54 believed that SOC is a relatively stable personality state, our findings show evidence that MBSR may influence SOC (i.e., to improve patients' ability to manage, comprehend, and finding meaning living with breast cancer).

Enhanced elements of mindfulness were shown for non‐reactivity and observing in the MBSR group. Future research is needed to explore the complexity and relations among the different dimensions of mindfulness, and to gain a deeper understanding about which factors facilitate the cultivation of mindfulness 69.

Our trial indicates that the benefits of MBSR may also extend to posttraumatic growth. Relatively little research has investigated the relationship between posttraumatic growth and immunity. A study of patients with hepatoma suggests that higher PTGI scores are associated with higher peripheral blood leukocytes and longer survival 70. Further research addressing the interrelationship of MBSR with posttraumatic growth and immune response is warranted.

In terms of biological response, changes in NK‐cell activity and numbers of both NK cells and B cells within the MBSR group as well as between groups were seen. Of note was the finding that there were no changes in numbers of IL‐6 or IL‐8. The clinical relevance of these discrete findings is difficult to estimate and more research is needed to fully explain the clinical meaning of these biological parameters. However, consistent with our findings, there is intriguing evidence suggesting that finding meaning and personal growth is associated with T‐cell levels 71 and NK‐cell activity 72. Furthermore, there have been prior reports that distress is associated with immune downregulation, including reduced NK‐cell activity 12, 32. In addition, participation in mindfulness training leads to a shift from proinflammatory response in cancer patients 33 and a pilot study has suggested that improvements in well‐being following MBSR was associated with increased NK activity and decreased CRP levels 73.

Given the beneficial efficacy of MBSR on both psychological and biological outcomes, future longitudinal studies may be needed to investigate the effect of these outcomes on disease progression and survival. There were several tendencies in favor for MBSR group, although not statistically significant, and for some outcome variables statistically significant changes could be detected only within MBSR group but not between the groups. A larger sample size might have resulted in more significant differences between groups. Several improvements were also seen in active controls (i.e., physical and social role functioning, and observing) as well as between groups (i.e., global distress, mental health, and posttraumatic growth). Future research is needed to explore who benefits from participating in an MBSR intervention with weekly group sessions versus using a self‐instructing training program.

This study is characterized by several strengths, including use of active and non‐MBSR controls, random assignment, inclusion of patient‐reported outcomes and immune response among a homogenous group of women diagnosed with breast cancer. To our knowledge, this is the first MBSR intervention study to include a comparison between standardized MBSR and both active controls using a self‐instructing program and passive controls. The notable study limitation was that all women who fulfilled the inclusion criteria were invited without undergoing screened for mood disorder before study invitation. Furthermore, despite randomization, there were differences in distribution regarding disease stage (tumor size and type of breast cancer) which might have affected study results.

In conclusion, results from this RCT suggest that MBSR is beneficial and leads to psychological and biological improvements. MBSR may hold potential for alleviating depression, distress and symptom experience, and to strengthen coping capacity, which may improve breast cancer survivorship. Since there were also positive changes in the active control group, it is important to provide the self‐training program to patients who prefer to practice themselves, without weekly group exercises. Finally, longitudinal studies are required to investigate whether these positive psychological and biological responses remain constant, increases or decreases over time.

## Conflict of Interest

The author(s) indicated no potential conflicts of interest.

## Supporting information


**Figure S1.** X. Typical result with dot plots showing CD3+, CD3+4+, CD3+8+, 19‐,and CD3‐16+56+ cells. Absolute numbers of cells were calculated using Trucount reference beads. Lymphocytes and Trucount beads were defined in a CD45 versus side scatter area (SSC‐A) and a CD19 versus SSC‐A plot, respectively. CD3+ and CD3‐ cells were then defined in a CD3 versus SSC‐A plot. Then CD3+4+ and CD3+8+ were defined in a plot of CD8 versus CD4. Finally CD19 and CD3‐16+56+ cells were defined in a plot of CD16+56+ versus CD19.
**Figure S2.** Y Typical result with dot plots showing regulatory T cells. Lymphocytes were defined in a forward scatter area (FSC‐A) versus side scatter area (SSC‐A) plot. CD3+4+ cells were then defined in a CD3 versus CD4 plot. Then CD3+4+25+ were defined in a plot of CD4 versus CD25. Finally CD3+4+25+FOXP3++ (regulatory T cells) cells were defined in a plot of CD25 versus FOXP3.
