# Supplementary material for: Mindfulness and its efficacy for psychological and biological responses in women with breast cancer
Source: Cancer Med. 2017 Apr 18;6(5):1108–22. doi: 10.1002/cam4.1052 (PMC5430085; doi:10.1002/cam4.1052)
Supplement: Supplementary file 1 — Figure S1. X. Typical result with dot plots showing CD3+, CD3+4+, CD3+8+, 19‐,and CD3‐16+56+ cells. Absolute numbers of cells were calculated using Trucount reference beads. Lymphocytes and Trucount beads were defined in a CD45 versus side scatter area (SSC‐A) and a CD19 versus SSC‐A plot, respectively. CD3+ and CD3‐ cells were then defined in a CD3 versus SSC‐A plot. Then CD3+4+ and CD3+8+ were defined in a plot of CD8 versus CD4. Finally CD19 and CD3‐16+56+ cells were defined in a plot of CD16+56+ versus CD19. Figure S2. Y Typical result with dot plots showing regulatory T cells. Lymphocytes were defined in a forward scatter area (FSC‐A) versus side scatter area (SSC‐A) plot. CD3+4+ cells were then defined in a CD3 versus CD4 plot. Then CD3+4+25+ were defined in a plot of CD4 versus CD25. Finally CD3+4+25+FOXP3++ (regulatory T cells) cells were defined in a plot of CD25 versus FOXP3. [file CAM4-6-1108-s001.pdf]

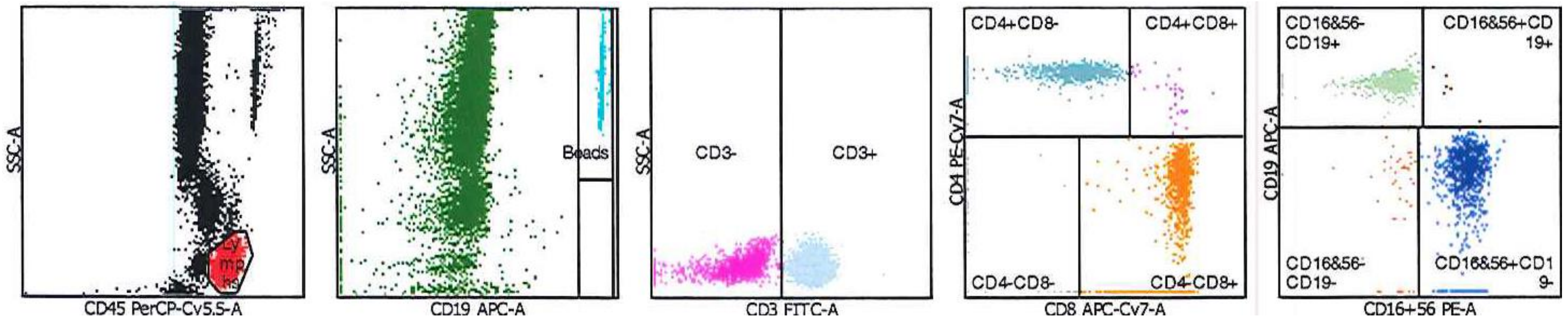

Fig X. Typical result with dot plots showing CD3+, CD3+4+, CD3+8+, 19- and CD3-16+56+ cells. Absolute numbers of cells were calculated using Trucount reference beads. Lymphocytes and Trucount beads were defined in a CD45 versus side scatter area (SSC-A) and a CD19 vesrsus SSC-A plot respectively. CD3+ and CD3- cells were then defined in a CD3 vesrus SSC-A plot. Then CD3+4+ and CD3+8+ were defined in a plot of CD8 versus CD4. Finally CD19 and CD3-16+56+ cells were defined in a plot of CD16+56+ versus CD19.

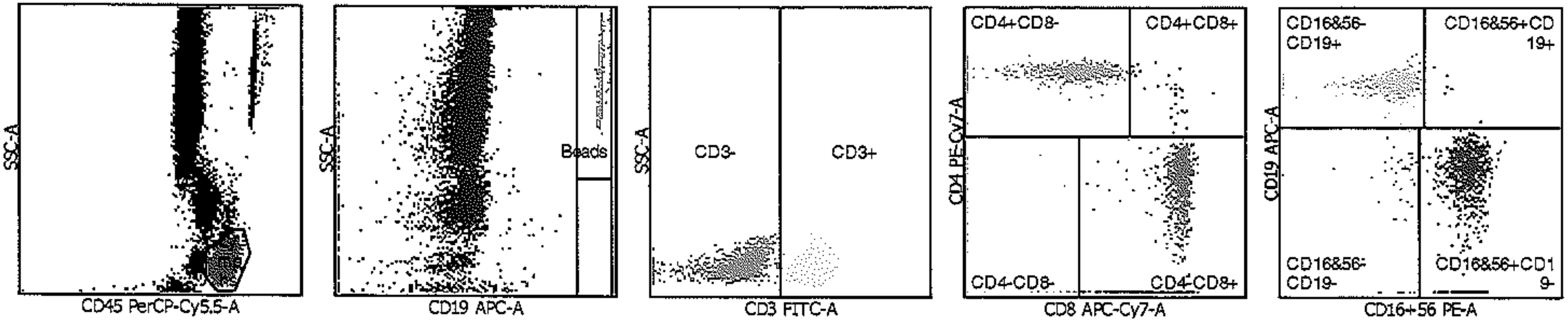

Fig X. Typical result with dot plots showing CD3+, CD3+4+, CD3+8+, 19- and CD3-16+56+ cells. Absolute numbers of cells were calculated using Trucount reference beads. Lymphocytes and Trucount beads were defined in a CD45 versus side scatter area (SSC-A) and a CD19 vesrsus SSC-A plot respectively. CD3+ and CD3- cells were then defined in a CD3 vesrus SSC-A plot. Then CD3+4+ and CD3+8+ were defined in a plot of CD8 versus CD4. Finally CD19 and CD3-16+56+ cells were defined in a plot of CD16+56+ versus CD19.

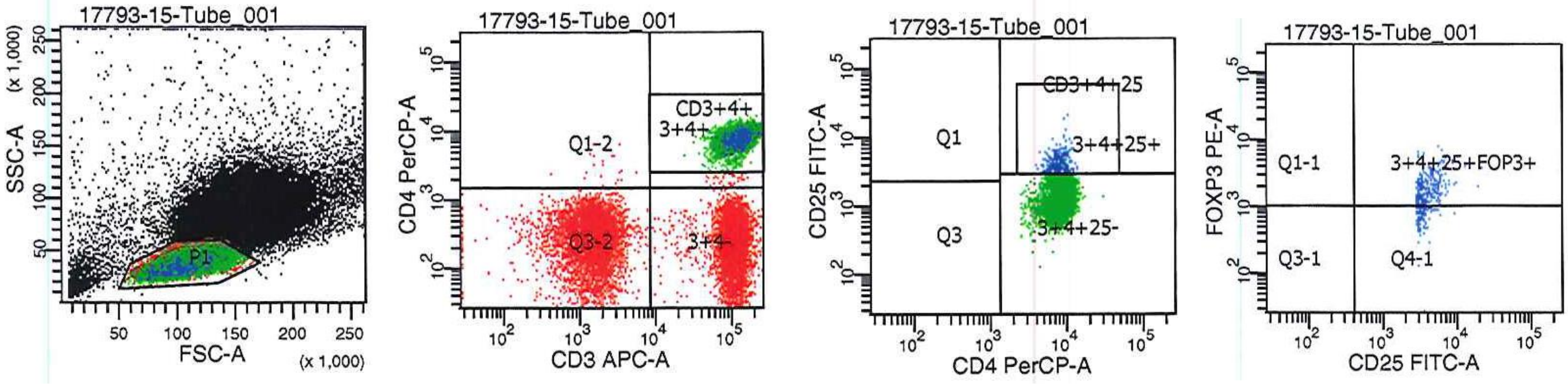

Fig Y. Typical result with dot plots showing regulatory T cells. Lymphocytes were defined in a forward scatter area (FSC-A) versus side scatter area (SSC-A) plot. CD3+4+ cells were then defined in a CD3 vesrus CD4 plot. Then CD3+4+25+ were defined in a plot of CD4 versus CD25. Finally CD3+4+25+FOXP3++ (regulatory T cells) cells were defined in a plot of CD25 versus FOXP3.

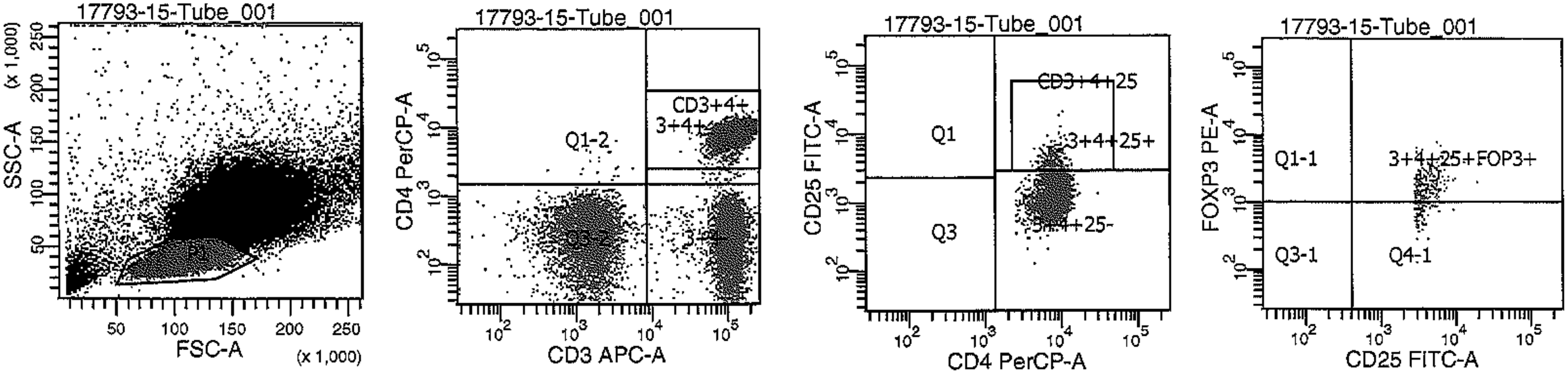

Fig Y. Typical result with dot plots showing regulatory T cells. Lymphocytes were defined in a forward scatter area (FSC-A) versus side scatter area (SSC-A) plot. CD3+4+ cells were then defined in a CD3 vesrus CD4 plot. Then CD3+4+25+ were defined in a plot of CD4 versus CD25. Finally CD3+4+25+FOXP3++ (regulatory T cells) cells were defined in a plot of CD25 versus FOXP3.
